# Engineering of the Photon Local Density of States: Strong Inhibition of Spontaneous Emission near the Resonant and High-Refractive Index Dielectric Nano-objects

**DOI:** 10.1021/acs.jpcc.1c09844

**Published:** 2022-03-16

**Authors:** Alina Muravitskaya, Artur Movsesyan, Dmitry V. Guzatov, Anne-Laure Baudrion, Pierre-Michel Adam, Sergey V. Gaponenko, Remi Vincent

**Affiliations:** † B.I. Stepanov Institute of Physics, National Academy of Sciences of Belarus, 68 Nezavisimosti Avenue, Minsk 220072, Belarus; ‡ Light, Nanomaterials & Nanotechnologies (L2n), CNRS EMR 7004, Université de Technologie de Troyes, 12 Rue Marie Curie, Troyes Cedex 10004, France; § Yanka Kupala State University of Grodno, str. Ozheshko 22, Grodno 230023, Belarus

## Abstract

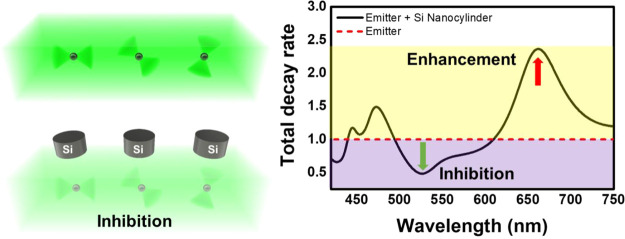

Metallic or dielectric nano-objects change the photon local density of states of closely placed emitters, particularly when plasmon or Mie resonances are present. Depending on the shape and material of these nano-objects, they may induce either a decrease or an increase in decay rates of the excited states of the emitter. In this work, we consider the reduction of the probability of optical transitions in emitters near high-refractive index dielectric (silicon and zinc selenide) nanoparticles. We tune the spectral positions of magnetic and electric modes of nanocylinders to obtain the largest overlap of the valleys in the total decay rate spectra for differently oriented dipoles and, in this way, find the highest inhibition of about 80% for randomly oriented emitters. The spectral positions of these valleys are easy to control since the wavelengths of the modes depend on the height and diameter of nanocylinders. The inhibition value is robust to the distance between the emitter and the nanoparticle in the range of nearly 50 nm, which is crucially important for the applications, such as selective optical transition engineering and photovoltaics.

## Introduction

The presence of a nanoparticle changes the decay rates of excited states of the molecules and atoms in its vicinity.^1^ Depending on the shape and material of the nanoparticle, it may induce either inhibition or enhancement of the relaxation processes and, correspondingly, increase or decrease the lifetime of excited states in these molecules or atoms. The enhancement of the spontaneous emission rate is of great interest for a wide range of applications, particularly in light-emitting devices, single-photon sources, and integrated photonics.^2−10^ Metal nanostructures are widely used in these applications due to the strong enhancement and high confinement of the optical near-fields under incident light illumination in resonance conditions and a consequent significant increase in the radiative decay.^3,9^ However, emitters near metal nanostructures also experience an increase in the nonradiative decay caused by the high absorption in metals. Therefore, the use of metal nanostructures demands careful control of the relative position of emitters and plasmonic nanoparticles.^10−13^ Recent studies show that the high-index dielectric nanoantennas are a highly promising alternative to metal nanoparticles in controlling the emission decay rates (often referred to as the photon local density of states, LDOS) because of the less-probable nonradiative relaxation channels and a greater variety of the excited modes and their easy control.^14−17^ In particular, dielectric nanoparticles support electric and magnetic resonances (Mie resonances) in the visible and near-infrared spectral range. The interplay of these modes results in highly promising properties such as enhanced electromagnetic fields, modification of the photon LDOS of emitters, nonlinear effects, metamaterial characteristics, and directional far-field coupling with low losses and low heating.^18−25^ Although this research field originated from mostly theoretical and simulation studies, at the moment, there are experimental reports of the dielectric nanoparticles used in enhanced Raman scattering and photoluminescence, dielectric Huygens’ metasurfaces, photonic topological insulators, and dielectric planar meta-optic devices.^19,21,23,26−28^ Also, the integration of the dielectric/semiconductor (for instance, silicon or gallium arsenide) nanoparticles into the nanophotonic and optoelectronic devices is straightforward due to the widespread use of these materials in the aforementioned applications.^21,23^

Inhibition of the spontaneous emission is of interest in the systems where it is beneficial to restrict the decay channels to only those that are necessary.^29,30^ For example, if the emitter has several emission channels, one of them can be suppressed to enhance the emission through the other ones.^31,32^ Also, in the case of photovoltaic or electronic devices, it is beneficial to decrease all radiative and nonradiative transitions. This effect was observed in photonic nanostructures with band gaps, where there are allowed and forbidden photonic bands.^29,33,34^ Then, when the emitter frequency is in the forbidden range, the spontaneous decay vanishes. The plasmonic and Mie resonance nanoantennas affect LDOS similarly. However, metal nanostructures under rare conditions can provide a limited decrease in the decay rates due to the high contribution of nonradiative decay channels.^14,30,35^ In contrast, the dielectric nanoantenna may be used for both enhancement and inhibition of decay processes, providing an additional control on the emission properties of a coupled system.^14,25,30,35^ The inhibition of the spontaneous emission was theoretically demonstrated for the fixed orientation of the dipole relative to a single Si nanosphere,^30,36,37^ a pair of Si nanospheres,^17^ and nanocylinders.^30,35^ The case of an averaged dipole orientation was characterized by only a slight lifetime increase (few percent), which was shown both in simulations and experimentally near Si nanocylinders.^35^ However, at the moment, the results are far from the inhibition reached in cavities or photonic crystals.^33,35,38^

In our previous work, we studied the increase in the lifetime of excited states induced by dielectric nanoparticles for their nonresonant spectral region.^30^ In this case, the inhibition was observed only for a dipole moment parallel to the nanoparticle surface, and therefore, it required control of the emitter orientation. Also, the spectral region of the inhibition was very broad due to the nonresonant character of the spectrum and the small size of the nanoparticles. In this work, we study the reduction of the emitters’ decay rate near Si nanoparticles in the spectral region of Mie resonances. We consider the excitation of high-order electric and magnetic modes and change their position to find the largest overlap between the areas without the modes. The spectral positions of these valleys depend on the height and diameter of nanocylinders. Our findings provide essential information for the engineering of the nanostructures with the precise control of the spectral windows of the enhancement or inhibition of spontaneous emission. First, we consider a simple case of a silicon nanosphere (NSp) and then nanocylinders (NCs) and their dimers.

## Methods

The modification of the total decay rate γ_total_/γ_0_ of an emitter (a molecule, a quantum dot, or an atom) placed near the nanoparticle can be defined as a relation of the power radiated in the vicinity of this nanoparticle to the power emitted in the free space in the absence of the particle.^39^ In the case considered in this article, we can write that
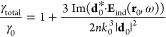
1where γ_0_ = 4*nk*_0_^3^|**d**_0_|^2^/(3ℏ) is a total decay rate in the absence of the nanoparticle; *n* is the refractive index of the dielectric medium surrounding the emitter; **d**_0_ is a dipole moment of the transition under consideration; **E**_ind_(**r**_0_,ω) is the induced electric field strength in the emitter position **r**_0_ and for the wavelength of emission ω; and *k*_0_ = ω/*c* = 2π/λ—wavenumber in a vacuum, where *c* is the speed of light and λ is a wavelength of light in vacuum. In the case of the nanostructure with absorption, the total decay rates appear as a sum of the radiative and nonradiative decay rates. When the emitter approaches the nanoparticle, the influence of the nonradiative decay rate increases, which induces a decrease in the lifetime of excited states and quenching of the emission. The scheme of the analytical calculations has been described in detail previously.^30,36,40^

The numerical calculations were performed using Lumerical FDTD based on the finite-difference time-domain (FDTD) method. We used the empirical dielectric constant retrieved from ref (41) for Si and ref (42) for ZnSe. The refractive index of the glass substrate (when used) was fixed at *n* = 1.5. Automatic nonuniform meshes were used with the refinement mesh of 1 nm around the nanoparticle and the dipole. As boundaries, perfectly matching layers were used. Symmetric or antisymmetric boundary conditions were chosen when they were applicable. The total power radiated by the dipole near the Si nanoparticle is evaluated including the part absorbed by the nanoantenna and radiative decay. This value was normalized to the radiation of the dipole in the absence of the nanoparticle. We calculated the averaged value between three orthogonal orientations
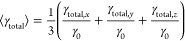
2where γ_total,*x*_, γ_total,*y*_, and γ_total,*z*_ are the total decay rates for the emitter oriented along the *X*, *Y*, and *Z* axis, respectively. *X* and *Y* orientations are called “tangential” in the text because in our spatial configuration, the dipole vector in these cases is parallel to the tangent line to a sphere or base of the nanocylinder. The *Z* orientation is called “normal” because it is perpendicular to the nanoparticle surface. Some of the maps are shown in the color saturation regime to underline the change of the total decay rate in the small intensity region below 1, which is smoothed out when the increase in the decay rates is much higher than 1.

## Results and Discussion

### Nanospheres

**Figure 1:**
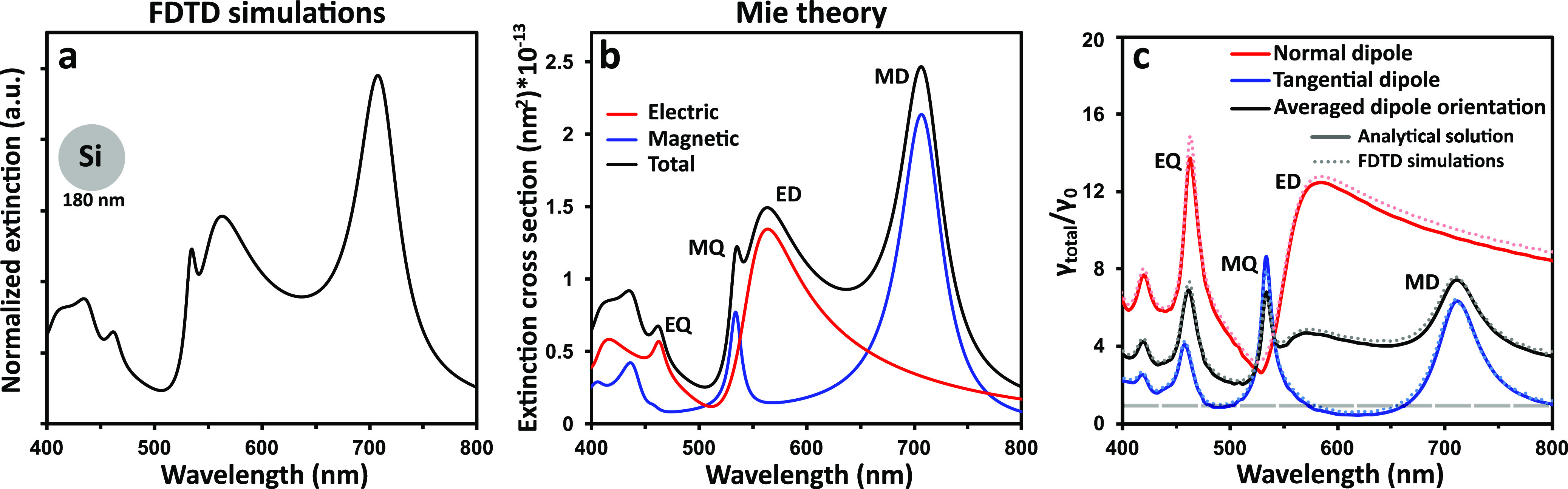
(a) Extinction spectrum of the 180 nm-diameter Si NSp in the air under the plane-wave excitation calculated by the FDTD method. (b) Extinction spectrum of 180 nm-diameter Si NSp in the air calculated using Mie theory (black curve). The contribution of the magnetic modes is plotted in blue, and the contribution of the electric modes is in red. MD—magnetic dipole, ED—electric dipole, MQ—magnetic quadrupole, and EQ—electric quadrupole. (c) Calculated relative total decay rate for an emitter placed 10 nm far from the 180 nm-diameter Si NSp for a dipole oriented normally (red curve) or tangentially (blue curve) to the sphere surface and the averaged case. The results of the analytical (solid lines) and numerical (dotted lines) approaches are compared. The dashed horizontal line corresponds to the constant γ_total_/γ_0_ = 1.

At first, we consider the simple case of a spherical dielectric nanoparticle with a high-refractive index (silicon, average value for the considered wavelength region *n* = 4.13). In Figure 1a, we present the extinction spectrum of a silicon NSp of 180 nm diameter placed in the air and under plane-wave illumination computed by the FDTD method. There are several peaks (modes) in the visible spectral range at 705, 565, 533, and 463 nm. Dielectric nanoparticles exhibit magnetic and electric modes such as dipolar, quadrupolar, and higher order modes.^36,43−45^ The excitation of the in-plane magnetic mode happens due to the coupling of the incident light with the displacement current loop along the height of the nanoparticle^18^ and therefore depends both on the height and lateral size of the nanostructure.^46^ At the same time, the electric modes are driven by the collective polarization of the material inside the nanoparticle along the oscillations of the incident light.^18^ For the precise determination of the nature of the excited modes, we perform the calculations based on Mie theory, where we can separate the contributions of magnetic and electric modes. Figure 1b shows the calculated extinction spectrum of the 180 nm-diameter Si NSp using Mie theory (black curve). The contributions of magnetic and electric modes are plotted by blue and red curves, respectively. The FDTD simulation and Mie calculation show a similar form of the spectra, and we can attribute all the excited modes as electrical and magnetic dipoles (ED and MD), quadrupoles (EQ and MQ), and octupoles.

In Figure 1c, we present the calculated relative total decay rate γ_total_/γ_0_ for an emitter placed 10 nm far from a Si NSp in the air. We consider two dipole orientations, either normal or tangential to the surface of the NSp. We used an analytic model described in refs (30)(36), and (40). Of important note is that the excitation of the electric or magnetic modes in dielectric nanoparticles depend on the orientation of the dipolar source relative to the nanoparticle surface.^36^ The dipole normally oriented to the surface strongly couples with the electric modes (ED and EQ) according to their spectral position determined by the red curve in Figure 1b, while the tangentially oriented dipole preferentially couples with the magnetic modes determined by the blue curve in Figure 1b. In the latter case, an electric contribution is also present; in particular, there are excited electric high-order modes. One may notice that γ_total_/γ_0_ is less than 1 (see the horizontal dashed line (γ_total_/γ_0_ = 1) in Figure 1c) in the spectral window at 570–650 nm for the tangential orientation of the dipole. It means that the decay process is inhibited due to the presence of the nanoparticle. The minima in the spectrum for the normally oriented dipole do not reach unity. The dip between the MQ and MD modes excited by the tangential dipole largely overlaps with the wide ED mode for the normal dipole orientation. Therefore, this overlap results in a flattened curve without inhibition areas for the averaged case (black curve in Figure 1c).

**Figure 2:**
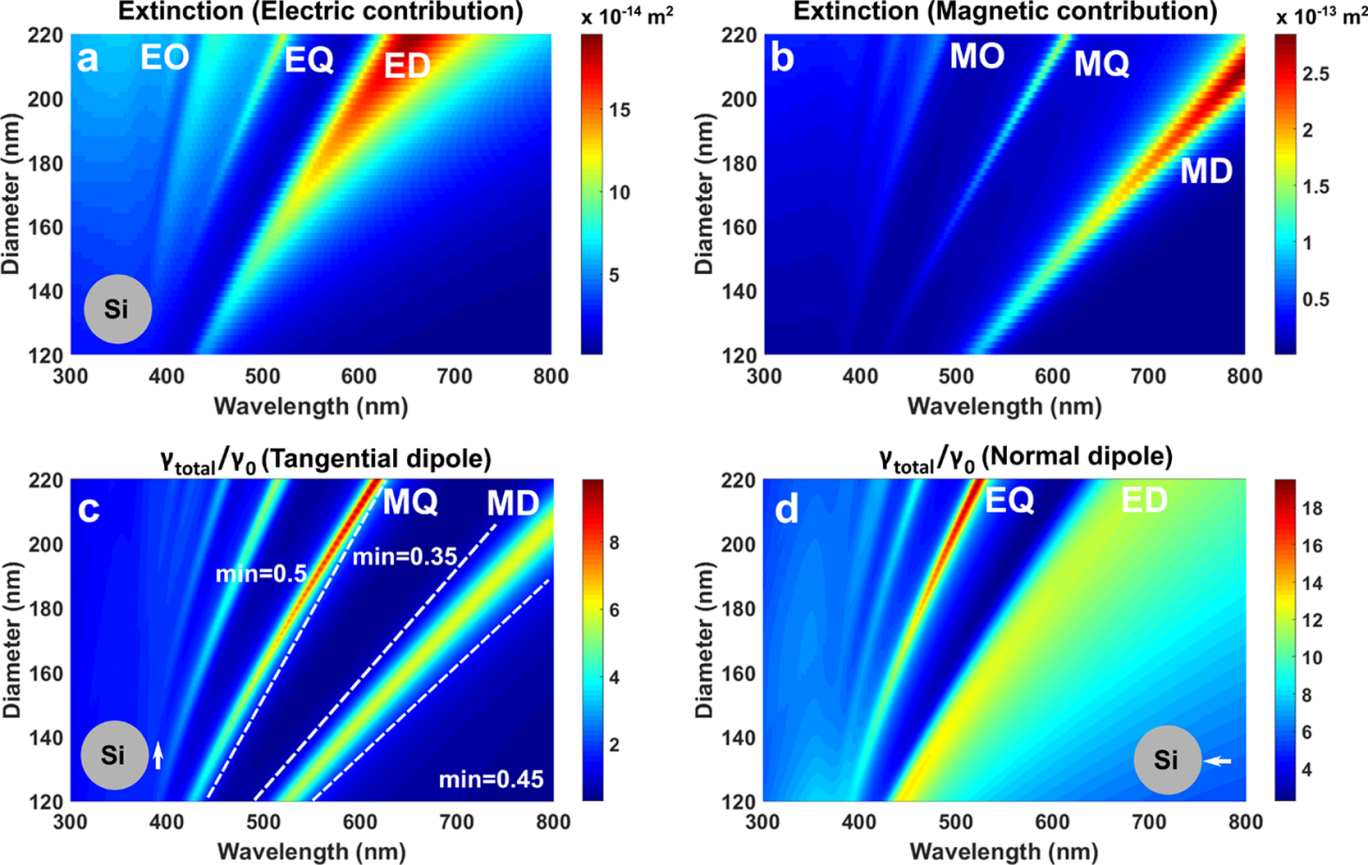
Extinction maps of Si nanospheres for electric (a) and magnetic (b) contributions and total decay rate maps for Si NSp excited by the tangential (c) and normal (d) dipoles placed 10 nm far from the sphere [marked modes: electrical and magnetic dipoles (ED, MD), quadrupoles (EQ, MQ), and octupoles (EO, MO)].

We calculated the extinction and total decay rate spectra for Si NSp of different diameters to follow the behavior of the electric and magnetic modes (Figure 2). The modes red-shift with an increase in the sphere’s diameter. The greatest increase in the decay rate occurs for the electrical modes (Figure 2d). The relative intensities of the dipolar and quadrupolar modes are different in the extinction and total decay spectra.^17,45^ The peaks in total decay (Figure 2c,d) associated with quadrupolar modes become more intensive than those of dipolar modes with the rise of the NSp size. One of the reasons is that we take into account the imaginary part of the dielectric permittivity of Si, and the increase in the total decay attributed to the higher order modes is largely connected with the nonradiative contribution. Interestingly, the inhibition happens in several spectral areas for the tangential dipole orientation, which are embraced by the dashed lines in Figure 2c (Figure S1a shows the map in the saturated regime). For the normal dipole orientation, γ_total_/γ_0_ does not go below unity, and the lowest values occur between two electric modes, ED and EQ (Figure 2d). The comparison of Figure 2c,d shows that the dips for one dipole orientation correspond to the maxima for the other dipole orientation, which limits the average inhibition for these structures.

**Figure 3:**
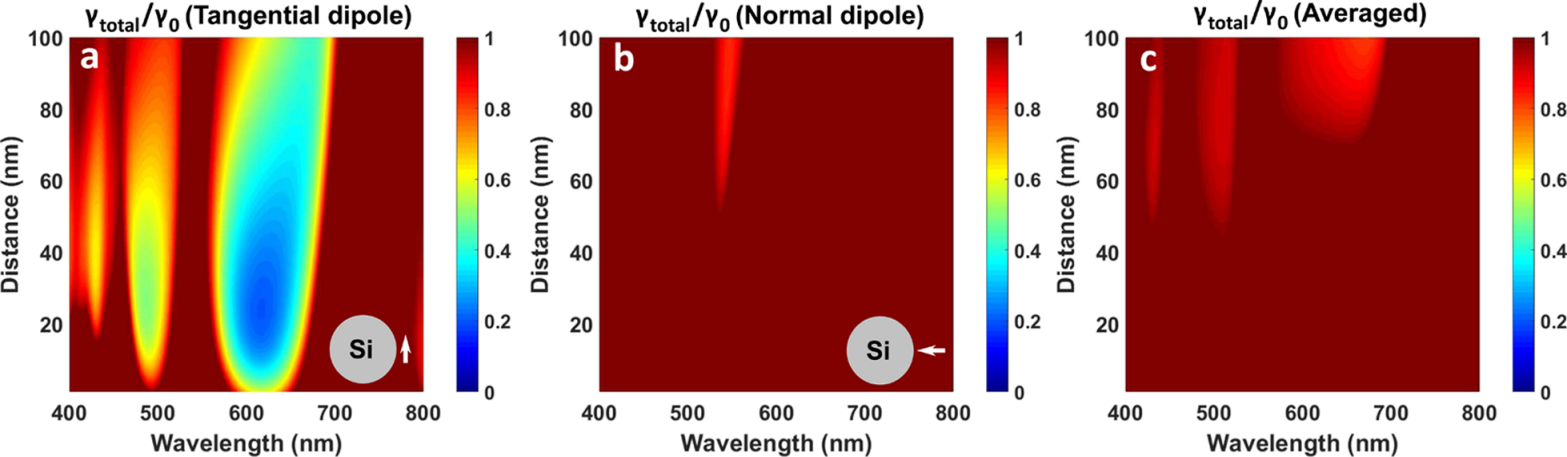
Total decay rate maps for the dipole [tangential (a), normal (b), and averaged (c) orientations relative to the nanosphere] placed at the different distances from 180 nm-diameter Si NSp. The maps are saturated to 1.

The inhibition or the enhancement of the decay rate highly depends on the distance between the emitter and the nanoparticle. In Figure 3a, we demonstrate the calculated total decay rate depending on the distance between the dipole oriented tangentially and the 180 nm-diameter NSp. We saturated the colorbar of the figure to 1 in order to observe where the inhibition happens. The largest inhibition windows occur at 580–650 nm and 480–500 nm. One may note that the optimal distance for the lowest decay rate is from 15 to 50 nm. For the normal dipole orientation (Figure 3b), 17% (γ_total_/γ_0_ = 0.83) inhibition is observed when the dipole is placed about 70 nm far from the NSp. At short distances, the emitter couples with both high radiative modes and also with optical losses to silicon nanoparticles and then has increased nonradiative decay rates. For the larger distances, interactions between the emitter and Si nanoparticles become weaker, and the inhibition also flattens out.

Figure 3c shows the total decay rate map for the averaged dipole orientation (⟨γ_total_⟩). There are several spectral windows where the total decay rate is below 1, about 0.85–0.92 (8–15% inhibition). According to the nonaveraged spectral maps (Figure 3a,b), inhibition happens mainly due to the large dips for the tangential orientation of the dipole. The electric modes are intensive, and their spectral position coincides with the inhibition areas between magnetic modes. As a result, overall inhibition regions are not broad, which is interesting for the spectrally selective inhibition for the emitters without a preferable orientation. However, the inhibition is strongly limited due to the large overlap of the valleys and peaks in the spectra for different dipole orientations.

### Nanocylinders

**Figure 4:**
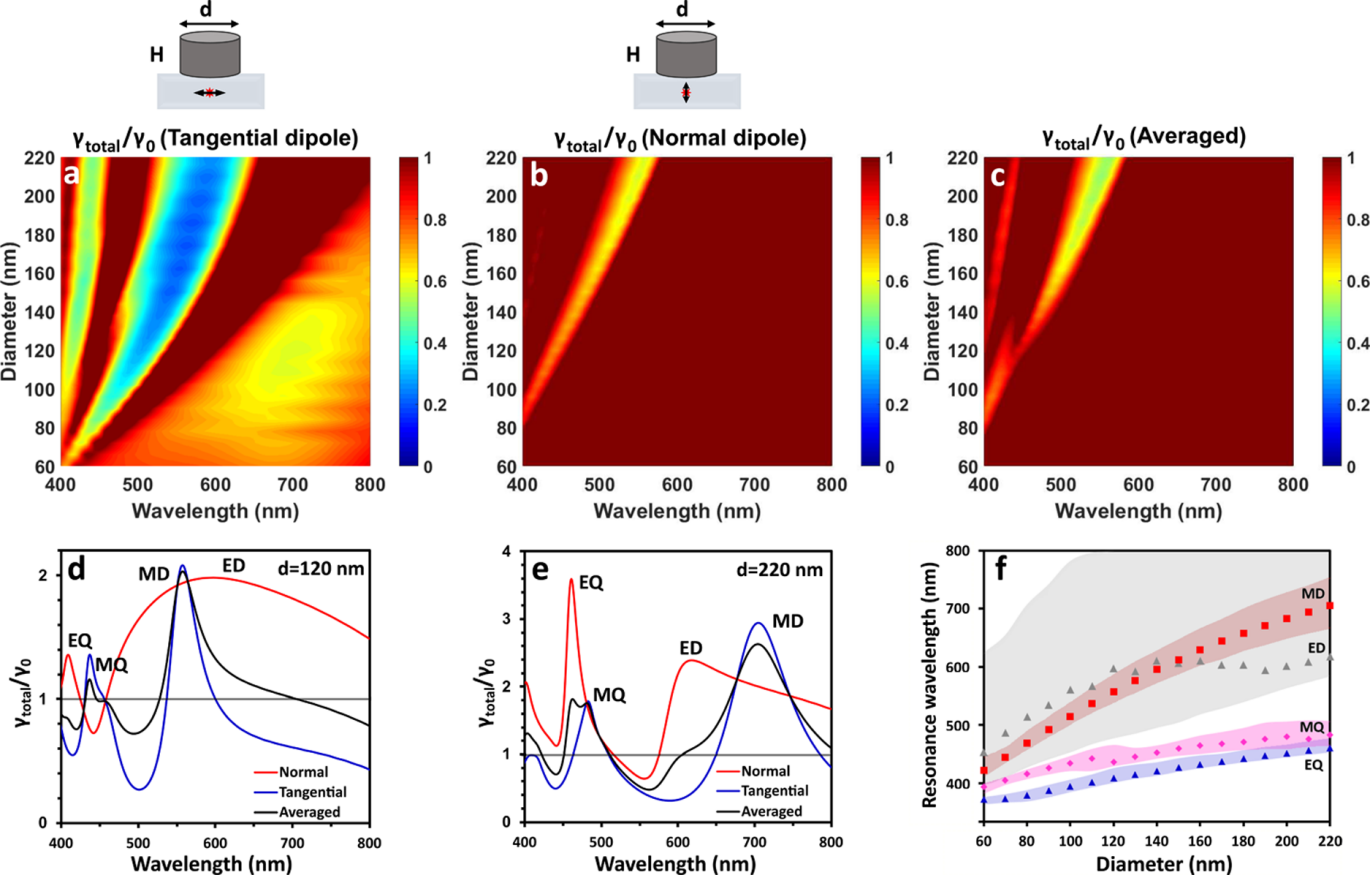
Total decay rate map for silicon nanocylinders of different diameters excited by (a) tangential or (b) normal dipoles and for averaged dipole orientation (c). The total decay rate for different dipole orientations near the Si NC of *d* = 120 nm (d) and *d* = 220 nm (e). Dependence of the electric and magnetic resonance positions on the radius of the Si NCs (the dots show the maximal positions and the areas show the half-maximal width) (f). The dipole is placed *r* = 35 nm far from the NC. The height of the NC is *H* = 110 nm.

To adjust the positions of the modes, we consider nanoparticles of a less-symmetrical shape such as Si nanocylinders (NCs) and vary their diameter–height ratio. The same approach was previously used to find the conditions for the efficient interference of the magnetic and electric modes, which resulted in a directional scattering.^46^ We also added a glass substrate to our model to simulate real experimental conditions. The nanocylinder in air was also considered in the Supporting Information (Figure S2). The fabrication of such “silicon-on-insulator” nanostructures is compatible with standard CMOS technology, which is widely spread in modern microelectronics and photonics.^47,48^ The emitter was embedded in a glass matrix to represent the experimental configuration (Figure 4 (scheme)); for instance, doped glasses with emitters are usually used as modules of DC (downconversion) and UC (upconversion). We consider NCs of height *H* = 110 nm. The emitter was placed on the distance of *r* = 35 nm. We chose this distance based on the values for the tangential dipole near the Si NSp (see Figure 3a).

Figure 4a,b demonstrates the dependence of the total decay rate for the tangential (parallel to the bottom plane of the NC) and normal (perpendicular to the bottom plane of the NC) dipole orientations. First, we compare the modes of nanocylinders (Figure S2a,b) and nanospheres (Figure 2c,d) in the air. The overall behavior of the modes is similar for the NSp and NC. Then, we make the attribution of the brightest modes present in the maps based on the previously reported results^18,36^ and similarities in the spectra for the NSp and NC of the aspect ratio 1. In particular, the NC of *H* = *D* = 110 nm (white dashed line in Figure S2a,b) corresponds in volume to the NSp of a diameter of 124 nm (Figure 2c,d). We found that the brightest modes for the tangential orientation are MD and MQ (532 and 426 nm for the NC and 535 and 432 nm for the NSp), and for the normal dipole orientation, the modes are ED and EQ (460 and 400 nm for the NC and 453 and 395 nm for the NSp). However, in the case of the nanosphere, the increase in the diameter (and therefore height) results in almost linear spectral shift of the magnetic and electric modes. For the NCs, the magnetic modes shift nonlinearly with an increase in the diameter, which allows changing the relative positions of the electric and magnetic modes.^46^ Due to this difference, cylindrical nanoparticles have a larger dip between electric and magnetic modes when the aspect ratio differs from unity. Important to note is that the maps are very similar in the case of the air surrounding (Figure S2a,c) and substrate (Figure 4a,c) due to the low refractive index of the substrate.^18^ For the tangential orientation of the dipole, the lowest values of 0.2–0.3 are in the valley between the MD and MQ, at 500–600 nm. The map for the normal dipole excitation has only one inhibition window (Figure 4b). This minimum between the ED and EQ shifts from 400 to 530 nm with the diameter increase. For the averaged dipole orientation (Figure 4c), the highest inhibition in the spectral region of 500–550 nm reaches 50%.

**Figure 5:**
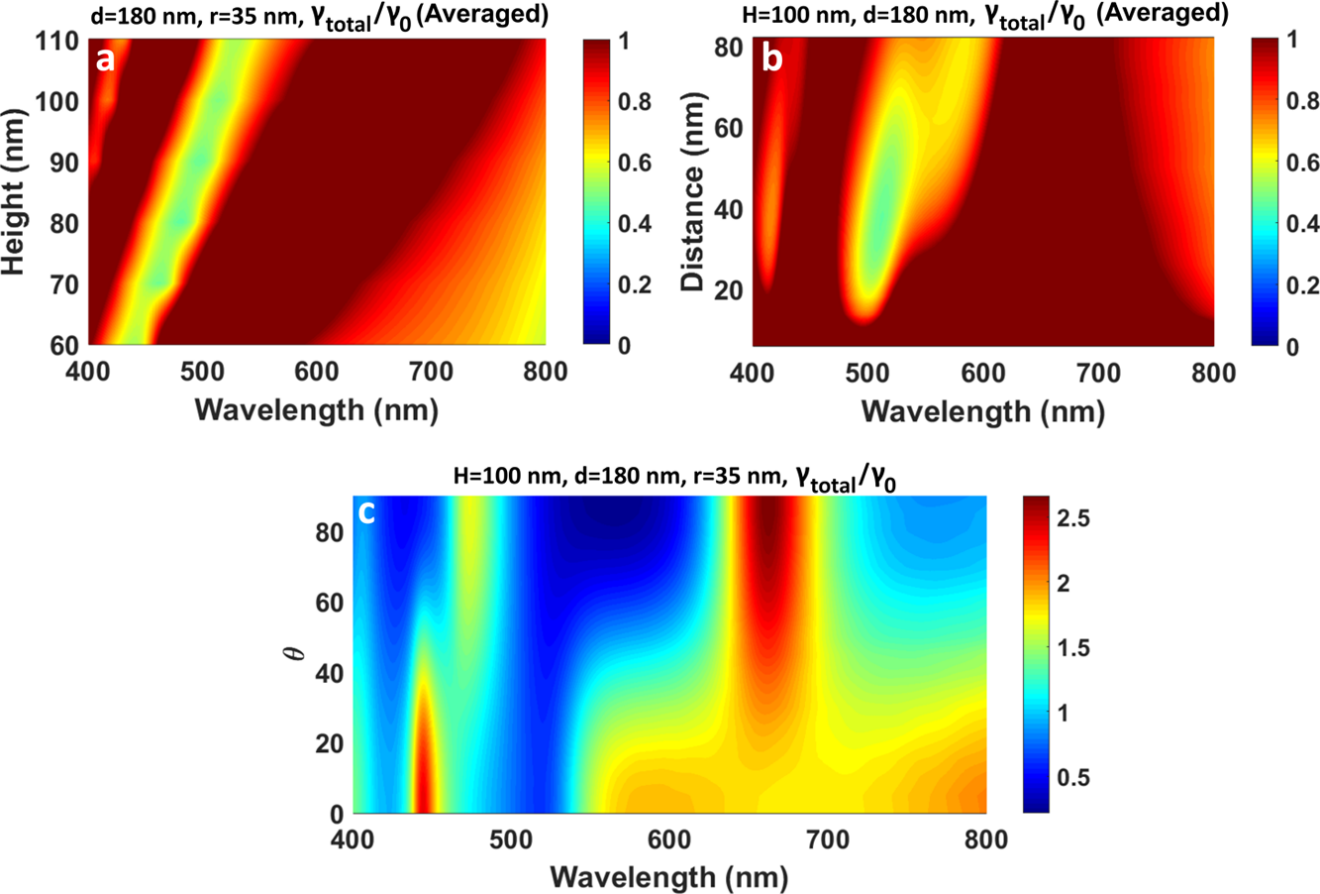
(a) Total decay rate map for a dipole (averaged orientation) near the 180 nm-diameter Si nanocylinders with different heights. The dipole is placed *r* = 35 nm far from the NC. (b) Total decay rate map for the dipole (averaged orientation) placed at different distances from the silicon NC (180 nm diameter and 100 nm height). The colorbar is saturated to 1 (nonsaturated, Figure S1b, is in the Supporting Information). The minimum value is ⟨γ_total_⟩ = 0.46. (c) Total decay rate map for a dipole with different orientations relative to the 180 nm-diameter and 100 nm-height Si NC (θ is an angle between the dipole and normal to the base of the NC).

We depicture the spectra for the nanocylinders of two sizes in Figure 4d,e to analyze the inhibition effect. One can see that for the smaller NC, the magnetic quadrupole is exactly at the place of the dip between the EQ and ED. This behavior is similar to the case of a NSp, and it can be explained to be close to the unit height–diameter ratio (*d* = 120 nm, *H* = 110 nm). For the NC of *d* = 220 nm, the modes are red-shifted, and both spectra for normal and tangential dipoles have valleys at 560 nm. In Figure 4f, we plotted the positions of all maxima depending on the size of the nanocylinders. We highlighted with a color the areas where the intensity of the resonances is higher than their half maxima. The modes of small NCs largely overlap with each other, but the spectral separation between them rises with the NC diameter increase, and the areas without excited modes expand. Due to this increase in the spectral resolution (spectral separation between modes), we can find inhibition areas for randomly oriented emitters. Important to note is that the position of this inhibition window can be changed by varying the sizes of nanocylinders. Figure 5a shows the dependence of the total decay rate spectra on the height of NC with a fixed diameter of 180 nm. The colorbar of the figure is saturated to 1. The position of the valley shifts from 430 to 510 nm with the increase in the height, whereas the minimum value stays almost constant for all sizes despite the size variation.

In the above-discussed calculations, the distance between the dipole and the nanocylinder was fixed at *r* = 35 nm. In order to verify the robustness of the inhibition dependence on the distance, we performed the numerical study presented in Figure 5b (the calculations for the NC in the air are presented in Figure S2d). It depicts the spectra for an emitter placed at different distances from the NC of 180 nm diameter and 100 nm height. One may note that the inhibition happens at 520 nm and for the distances from 15 nm. Moreover, the variation of the intensity of the inhibition is low for the distances of 35 nm < *r* < 70 nm. For the larger distances, the modification of the LDOS tends to disappear.

Importantly, nanocylinders feature stronger and wider inhibition areas than nanospheres for the dipole sources averaged over three orthogonal axes (Figure 3c). For the NSp, the dips in the spectra for the tangential orientation are almost compensated by the electric modes excited in the same spectral range by the dipole of other orientation. Therefore, the suppression of the decay is limited and appears only at larger distances of more than 60 nm where all the modes are not intensive. In the case of the NCs, we tune the sizes of nanoantennas to achieve a large overlap of the dips in the spectra for the tangential and normal orientations of the dipole. Moreover, a selective variation of the nanocylinder diameter or height results in an overall greater total decay decrease in the areas in between modes. One can expect similar behavior for the other nonsymmetrical nanostructures such as ellipsoids or prisms. The largest overlap happens at 520 nm, and since no modes are excited in this area, the decay rate falls fast with an increase in the distance between the dipole and NC. The dip at 560 nm is mostly pronounced in the tangential dipole spectra, and then, it appears for the averaged case only for the distance of more than 50 nm due to the impact of the modes excited by the normally oriented dipole. Such behavior is beneficial for multiple applications since there is no need for precise control of the emitter position and orientation. Furthermore, the inhibition can be obtained for multiple layers of emitters. For example, this configuration can be used for the simultaneous inhibition of the transitions for the wavelengths between 510 and 520 nm (average value for 35 nm < *r* < 70 nm is ⟨γ_total_⟩_*r*_ = 0.54) and promotion of the decay paths for the spectral regions 630–640 nm (average value for 35 nm < *r* < 70 nm is ⟨γ_total_⟩_*r*_ = 2) (Figure S1b).

Here, we calculated and analyzed the averaged value for three orthogonal orientations, which corresponds to the incoherent unpolarized dipole or ensemble of the emitters without a determined orientation near the nanoantenna (the interactions between the emitters are neglected). This case is one of the most straightforward in an experimental realization, but our findings suggest that the inhibition should not depend largely on the dipole orientation since we overlap the dips in the spectra for the normal and tangential dipoles. Figure 5c shows the dependence of the total decay rate on the orientation of the dipole vector relative to the normal to the base of the nanocylinder. One can see how the electric modes for the normal dipole gradually change to the magnetic modes for the tangential dipole orientation. All of them are pronounced in the middle case between 30 and 60°. However, all curves have minima in the spectral region of 500–530 nm. The lowest variation appears at 506 nm where all orientations feature the inhibition value of 0.70. This wavelength corresponds to the crossing of the spectra for the normal and tangential orientations of the dipole but is offset from the minimum position for the averaged dipole orientation. At 520 nm, the dip value varies from 0.35 (θ = 90°) to 0.69 (θ = 0°), which results in the lowest averaged value of ⟨γ_total_⟩ = 0.46 between three orientations (two tangential and one normal). Therefore, we found conditions for the total decay rate inhibition down to 30% for an individual dipole of any orientation and even stronger averaged inhibition for the randomly oriented ensemble of emitters.

Similar properties can be observed for dielectric nanoparticles made of other materials of high refractive index. As an example, we consider ZnSe nanocylinders placed on a glass substrate. ZnSe is a widely used semiconductor material with low absorption and a relatively high refractive index (average value for the considered wavelength region *n* = 2.68),^49^ which is the preferred material for optical components such as lenses and beam expanders. Also, there is an expansive range of ZnSe-based quantum dots emitting in a wide spectral region.^50−52^ The fabrication of the ZnSe nanostructures^53^ and their Mie resonances^54^ were explored as well and applied in the surface-enhanced Raman scattering^54,55^ and photocatalysis.^56^Figure S3a shows the calculated total decay rate for a dipole beneath the ZnSe nanocylinder of 180 nm height and different diameters. The emitter is embedded in the substrate. One can see that the minima are in the areas between the electric dipole and other high-order modes. The value of ⟨γ_total_⟩_*r*_ = 0.6 is reached at 410–450 nm for the diameters of 180–220 nm. We chose the distance between the NC and the emitter of *r* = 35 nm due to the previous results for the Si nanocylinders, and when we map the dependence of the total decay rate on the distance (Figure S3b), we find that the minimum is indeed in this area. The inhibition occurs for a wide range of distances between the emitter and the nanocylinder. It starts at around 10 nm and continues until 80 nm. The increase in the decay rate is observed around 480–490 nm. These results show the case study for ZnSe nanocylinders and also prove the concept of the selective inhibition of relaxation processes with the use of semiconductor/dielectric nanoparticles.

### Vertical Dimers

**Figure 6:**
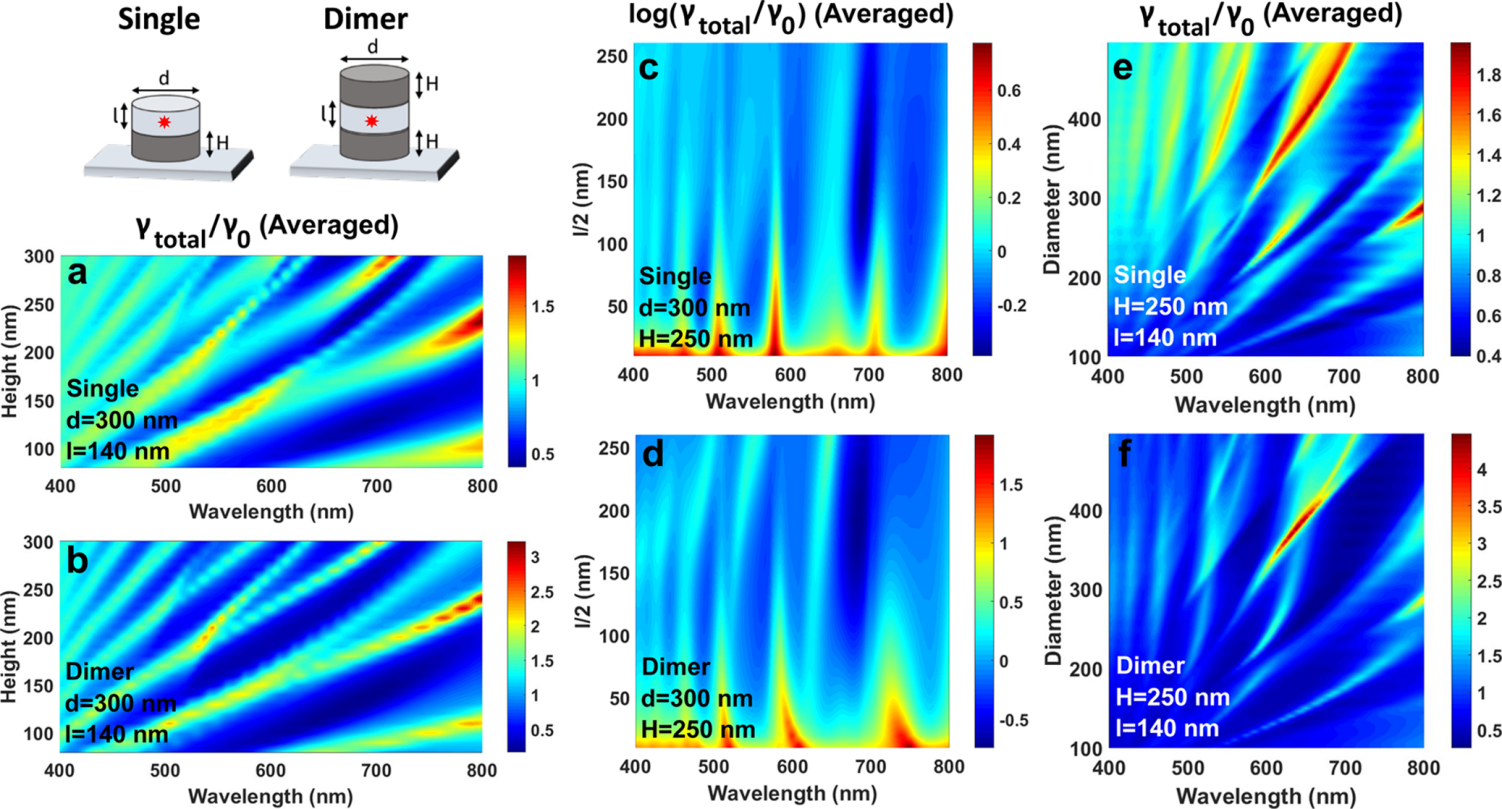
Total decay rate maps for the dipole (averaged orientations) on top of the single silicon NC (a,c,d) or in the middle of the dimer of silicon NCs (*d* = 300 nm) (b,d,e) on the substrate. A dipole is embedded in the middle of the dielectric spacer disk (scheme at the top left of the figure). (a,b) Height of the NCs is varied. The diameter is *d* = 300 nm; the thickness of the spacer layer is *l* = 140 nm. (c,d) Distance between the dipole and nanocylinders is varied by the increase in the spacer dielectric disk. The NC diameter is *d* = 300 nm; the height of the NCs is *H* = 250 nm. The maps are presented in the logarithm scale (the inhibition is, then, below 0). (e,f) Diameter of the NCs is varied. The fixed parameters are *H* = 250 nm and *l* = 140 nm.

The above-considered nanostructures have limited sizes to study the behavior of the dipolar and quadrupolar modes and understand the conditions for the most effective inhibition. Next, we broaden the range of the NC sizes to manipulate the higher number of the excited modes and also consider the vertical dimer of NCs separated by a thin disk of a lower refractive index (*n* = 1.5). The emitter was placed inside this thin disk. These nanostructures can be fabricated by the lithographical methods and as shown previously^16,57−59^ may have different behaviors in comparison with monomer structures. To compare the monomer and dimer cases, we put the emitter inside the dielectric disk (*n* = 1.5) on top of the monomer (Figure 6, scheme), which corresponds to the configuration in sensing experiments.

One can see in Figure 6a,b that for NCs of 300 nm diameter, we can control the position of the minima in the wider wavelength range from 550 to 800 nm. The increase in the NC sizes results in the excitation of higher order modes, and we can observe multiple minimal areas. The main difference between monomer (Figure 6a) and dimer (Figure 6b) cases manifests itself in the relative intensity of the modes. The minimum values are ⟨γ_total_⟩ = 0.17 for the dimer and ⟨γ_total_⟩ = 0.41 for the monomer. Also, the maximum decay rate enhancement is higher for the dimer.

The dependences of the total decay rate on the thickness of the thin disk (distance between the dipole and the NC) are also similar for the dimer and monomer (Figure 6c,d). For this case, we present the maps in the logarithmic scale since the difference between the minima and maxima is of two orders of magnitude. The inhibition occurs at a disk thickness of 80 < *l* < 260 nm, which corresponds to the distance between the NC and dipole of 40 nm < *l*/2 < 130 nm. The expansion of the inhibition distances relative to the small NC (Figure 5b) is mainly connected with the increased size of the nanoantenna and, therefore, stronger interactions with the dipole. The average values at 690 nm are ⟨γ_total_⟩_*r*_ = 0.5 for the monomer and ⟨γ_total_⟩_*r*_ = 0.23 for the dimer for the 40 nm < *l*/2 < 120 nm region. The total enhancement of the decay rate for the interparticle distance of 10 nm reaches 80-fold (1.9 in the logarithmic scale) in the case of the dimer and 6-fold for the monomer. A similar level of enhancement was previously observed for the dimer of Si nanospheres with the same interparticle distance of 10 nm.^17^ Finer tuning of the dip position and intensity can be performed by varying the diameter of NCs (Figure 6e,f). These maps show a high variety of the excited modes in these structures. The difference between enhancement and inhibition is more pronounced in the case of the dimer. For instance, the enhancement of the decay rate reaches ⟨γ_total_⟩ = 4.5 at 632 nm for the dimer of NCs (*d* = 380 nm), and the inhibition for the same structure at 700 nm is ⟨γ_total_⟩ = 0.3. Note that in the case of the dimer, the inhibition for the parallel dipole orientation is higher than for the case of a single NC (not shown here). However, this decrease is compensated by the rise in the radiative decay for the normal dipole orientation. A similar difference between these two orientations of a dipole in dimer structures was previously demonstrated for the other shapes and materials.^16,57−59^

## Conclusions

In conclusion, we found the conditions for the strong inhibition of the decay rate for randomly oriented emitters near the high-refractive index dielectric nanoparticles. The inhibition value is robust to the distance between the emitter and the nanoparticle in the range of nearly 50 nm, which is crucially important for the applications. We tuned the spectral positions of magnetic and electric modes of high-refractive index nanocylinders to obtain the largest overlap of the valleys in the decay rate spectra for the differently oriented dipoles and, in this way, found the highest inhibition ever reported for randomly oriented emitters near dielectric nanoparticles. In particular, we demonstrated the total decay inhibition down to 30% for an individual dipole of any orientation and even stronger averaged inhibition for the randomly oriented ensemble of emitters. Such selective control of the local density of optical states can help manipulate the emission color of an emitter with several relaxation channels and induce redistribution of the probability of various internal radiative and nonradiative pathways. Also, the inhibition can be used in a selective detection of a fluorescent molecule in the mixture of different fluorophores. For instance, the dielectric nanoparticle can suppress the unnecessary emission channels, whereas the fluorescence of a molecule of interest is enhanced. Also, the effect can provide the selective enhancement of the anti-Stokes versus Stokes Raman scattering due to the contribution in this phenomenon of the photon local density of states.^60^

## Supporting Information Available

Calculated total decay rate maps for the dipole (averaged orientations) placed 10 nm far from the Si nanosphere and at different distances from the silicon nanocylinder in the air and on substrate and calculated total decay rate map for the dipole (averaged orientations) near the ZnSe nanocylinder (PDF)

## References

[ref1] Purcell E. M. Spontaneous Emission Probabilities at Radio Frequencies. Phys. Rev. 1946, 340, 839 10.1007/978-1-4615-1963-8_40.

[ref2] Ozbay E. Plasmonics: Merging Photonics and Electronics at Nanoscale Dimensions. Science 2006, 311, 189–193. 10.1126/science.1114849. 16410515

[ref3] Gaponenko S. V.; Demir H. V. Applied Nanophotonics; Cambridge University Press: Cambridge, U.K., 2018.

[ref4] Akselrod G. M.; Argyropoulos C.; Hoang T. B.; Ciracì C.; Fang C.; Huang J.; Smith D. R.; Mikkelsen M. H. Probing the Mechanisms of Large Purcell Enhancement in Plasmonic Nanoantennas. Nat. Photonics 2014, 8, 835–840. 10.1038/nphoton.2014.228.

[ref5] Lozano G.; Rodriguez S. R.; Verschuuren M. A.; Gómez Rivas J. Metallic Nanostructures for Efficient LED Lighting. Light: Sci. Appl. 2016, 5, e16080 10.1038/lsa.2016.80. 30167168 PMC6059959

[ref6] Park H. C.; Isnaeni; Gong S.; Cho Y. H. How Effective Is Plasmonic Enhancement of Colloidal Quantum Dots for Color-Conversion Light-Emitting Devices?. Small 2017, 13, 1701805 10.1002/smll.201701805. 29120086

[ref7] Movsesyan A.; Lamri G.; Kostcheev S.; Horneber A.; Bräuer A.; Meixner A. J.; Fleischer M.; Zhang D.; Baudrion A.-L.; Adam P.-M. Enhanced Two-Photon Photoluminescence Assisted by Multi-Resonant Characteristics of a Gold Nanocylinder. Nanophotonics 2020, 9, 4009–4019. 10.1515/nanoph-2020-0213.

[ref8] Perveen A.; Deng L.; Muravitskaya A.; Yang D.; Movsesyan A.; Gaponenko S.; Chang S.; Zhong H. Enhanced Emission of In-Situ Fabricated Perovskite-Polymer Composite Films on Gold Nanoparticle Substrates. Opt. Mater. Express 2020, 10, 1659–1674. 10.1364/ome.10.001659.

[ref9] Koenderink A. F. Single-Photon Nanoantennas. ACS Photonics 2017, 4, 710–722. 10.1021/acsphotonics.7b00061. 29354664 PMC5770162

[ref10] Jeong Y.; Kook Y.-M.; Lee K.; Koh W.-G. Metal Enhanced Fluorescence (MEF) for Biosensors: General Approaches and a Review of Recent Developments. Biosens. Bioelectron. 2018, 111, 102–116. 10.1016/j.bios.2018.04.007. 29660581

[ref11] Gaponenko S. V.; Guzatov D. V. Colloidal Plasmonics for Active Nanophotonics. Proc. IEEE 2020, 108, 704–720. 10.1109/jproc.2019.2958875.

[ref12] Bauch M.; Toma K.; Toma M.; Zhang Q.; Dostalek J. Plasmon-Enhanced Fluorescence Biosensors: A Review. Plasmonics 2014, 9, 781–799. 10.1007/s11468-013-9660-5. 27330521 PMC4846700

[ref13] Trotsiuk L.; Muravitskaya A.; Kulakovich O.; Guzatov D.; Ramanenka A.; Kelestemur Y.; Demir H. V.; Gaponenko S. Plasmon-Enhanced Fluorescence in Gold Nanorod-Quantum Dot Coupled Systems. Nanotechnology 2020, 31, 105201 10.1088/1361-6528/ab5a0e. 31751975

[ref14] Bidault S.; Mivelle M.; Bonod N. Dielectric Nanoantennas to Manipulate Solid-State Light Emission. J. Appl. Phys. 2019, 126, 094104 10.1063/1.5108641.

[ref15] Evlyukhin A. B.; Novikov S. M.; Zywietz U.; Eriksen R. L.; Reinhardt C.; Bozhevolnyi S. I.; Chichkov B. N. Demonstration of Magnetic Dipole Resonances of Dielectric Nanospheres in the Visible Region. Nano Lett. 2012, 12, 3749–3755. 10.1021/nl301594s. 22703443

[ref16] Rolly B.; Bebey B.; Bidault S.; Stout B.; Bonod N. Promoting Magnetic Dipolar Transition in Trivalent Lanthanide Ions with Lossless Mie Resonances. Phys. Rev. B 2012, 85, 245432 10.1103/physrevb.85.245432.

[ref17] Albella P.; Poyli M. A.; Schmidt M. K.; Maier S. A.; Moreno F.; Sáenz J. J.; Aizpurua J. Low-Loss Electric and Magnetic Field-Enhanced Spectroscopy with Subwavelength Silicon Dimers. J. Phys. Chem. C 2013, 117, 13573–13584. 10.1021/jp4027018.

[ref18] van de Groep J.; Polman A. Designing Dielectric Resonators on Substrates: Combining Magnetic and Electric Resonances. Opt. Express 2013, 21, 26285–26302. 10.1364/oe.21.026285. 24216852

[ref19] Kruk S.; Kivshar Y. Functional Meta-Optics and Nanophotonics Govern by Mie Resonances. ACS Photonics 2017, 4, 2638–2649. 10.1021/acsphotonics.7b01038.

[ref20] Cihan A. F.; Curto A. G.; Raza S.; Kik P. G.; Brongersma M. L. Silicon Mie Resonators for Highly Directional Light Emission from Monolayer MoS_2_. Nat. Photonics 2018, 12, 284–290. 10.1038/s41566-018-0155-y.

[ref21] Paniagua-Dominguez R.; Ha S. T.; Kuznetsov A. I. Active and Tunable Nanophotonics with Dielectric Nanoantennas. Proc. IEEE 2020, 108, 749–771. 10.1109/jproc.2019.2943183.

[ref22] Xu J.; Wu Y.; Zhang P.; Wu Y.; Vallée R. A. L.; Wu S.; Liu X. Resonant Scattering Manipulation of Dielectric Nanoparticles. Adv. Opt. Mater. 2021, 9, 2100112 10.1002/adom.202100112.

[ref23] Staude I.; Pertsch T.; Kivshar Y. S. All-Dielectric Resonant Meta-Optics Lightens Up. ACS Photonics 2019, 6, 802–814. 10.1021/acsphotonics.8b01326.

[ref24] Capretti A.; Lesage A.; Gregorkiewicz T. Integrating Quantum Dots and Dielectric Mie Resonators: A Hierarchical Metamaterial Inheriting the Best of Both. ACS Photonics 2017, 4, 2187–2196. 10.1021/acsphotonics.7b00320. 29057294 PMC5646587

[ref25] Hasan M. R.; Hellesø O. G. Dielectric Optical Nanoantennas. Nanotechnology 2021, 32, 202001 10.1088/1361-6528/abdceb. 33461187

[ref26] Dmitriev P. A.; Baranov D. G.; Milichko V. A.; Makarov S. V.; Mukhin I. S.; Samusev A. K.; Krasnok A. E.; Belov P. A.; Kivshar Y. S. Resonant Raman Scattering from Silicon Nanoparticles Enhanced by Magnetic Response. Nanoscale 2016, 8, 9721–9726. 10.1039/c5nr07965a. 27113352

[ref27] Lin D.; Fan P.; Hasman E.; Brongersma M. L. Dielectric Gradient Metasurface Optical Elements. Science 2014, 345, 298–302. 10.1126/science.1253213. 25035488

[ref28] Yuan S.; Qiu X.; Cui C.; Zhu L.; Wang Y.; Li Y.; Song J.; Huang Q.; Xia J. Strong Photoluminescence Enhancement in All-Dielectric Fano Metasurface with High Quality Factor. ACS Nano 2017, 11, 10704–10711. 10.1021/acsnano.7b04810. 29023088

[ref29] Yablonovitch E. Inhibited Spontaneous Emission in Solid-State Physics and Electronics. Phys. Rev. Lett. 1987, 58, 2059–2062. 10.1103/physrevlett.58.2059. 10034639

[ref30] Gaponenko S. V.; Adam P.-M.; Guzatov D. V.; Muravitskaya A. O. Possible Nanoantenna Control of Chlorophyll Dynamics for Bioinspired Photovoltaics. Sci. Rep. 2019, 9, 7138 10.1038/s41598-019-43545-4. 31073157 PMC6509350

[ref31] Chen G.; Li X. Tuning the Emission Color of a Quantum Emitter by Photonic Local Density of States. Opt. Lett. 2021, 46, 2750–2753. 10.1364/ol.423589. 34061104

[ref32] Gao T.; Zhu X.; Wu X. J.; Zhang B.; Liu H. L. Selectively Manipulating Upconversion Emission Channels with Tunable Biological Photonic Crystals. J. Phys. Chem. C 2021, 125, 732–739. 10.1021/acs.jpcc.0c08636.

[ref33] Yablonovitch E. Photonic Band-Gap Structures. J. Opt. Soc. Am. B 1993, 10, 283 10.1364/josab.10.000283.

[ref34] Gaponenko S. V. Introduction to Nanophotonics; Cambridge University Press: Cambridge, U.K., 2010.

[ref35] Bouchet D.; Mivelle M.; Proust J.; Gallas B.; Ozerov I.; Garcia-Parajo M. F.; Gulinatti A.; Rech I.; De Wilde Y.; Bonod N.; et al. Enhancement and Inhibition of Spontaneous Photon Emission by Resonant Silicon Nanoantennas. Phys. Rev. Appl. 2016, 6, 064016 10.1103/physrevapplied.6.064016.

[ref36] Schmidt M. K.; Esteban R.; Sáenz J. J.; Suárez-Lacalle I.; Mackowski S.; Aizpurua J. Dielectric Antennas – a Suitable Platform for Controlling Magnetic Dipolar Emission. Opt. Express 2012, 20, 18609 10.1364/oe.20.018609. 22714428

[ref37] Krasnok A. E.; Slobozhanyuk A. P.; Simovski C. R.; Tretyakov S. A.; Poddubny A. N.; Miroshnichenko A. E.; Kivshar Y. S.; Belov P. A. An Antenna Model for the Purcell Effect. Sci. Rep. 2015, 5, 12956–16. 10.1038/srep12956. 26256529 PMC4530337

[ref38] Hulet R. G.; Hilfer E. S.; Kleppner D. Inhibited Spontaneous Emission by a Rydberg Atom. Phys. Rev. Lett. 1985, 55, 2137–2140. 10.1103/physrevlett.55.2137. 10032058

[ref39] Novotny L.; Hecht B. Principles of Nano-Optics; Cambridge University Press: Cambridge, U.K., 2006.

[ref40] Guzatov D. V.; Vaschenko S. V.; Stankevich V. V.; Lunevich A. Y.; Glukhov Y. F.; Gaponenko S. V. Plasmonic Enhancement of Molecular Fluorescence near Silver Nanoparticles: Theory, Modeling, and Experiment. J. Phys. Chem. C 2012, 116, 10723–10733. 10.1021/jp301598w.

[ref41] Palik E. D. Handbook of Optical Constants of Solids; Academic Press, 1985.

[ref42] Amotchkina T.; Trubetskov M.; Hahner D.; Pervak V. Characterization of E-Beam Evaporated Ge, YbF_3_, ZnS, and LaF_3_ Thin Films for Laser-Oriented Coatings. Appl. Opt. 2020, 59, A40 10.1364/ao.59.000a40. 32225351

[ref43] Movsesyan A.; Besteiro L. V.; Wang Z.; Govorov A. O. Mie Sencing with Neural Network: Recognition of Nano-Object Parameters, the Invisibility Point, and Restricted Models. Adv. Theory Simul. 2022, 5, 2100369 10.1002/adts.202100369.

[ref44] Chaâbani W.; Proust J.; Movsesyan A.; Béal J.; Baudrion A.-L.; Adam P.-M.; Chehaidar A.; Plain J. Large-Scale and Low-Cost Fabrication of Silicon Mie Resonators. ACS Nano 2019, 13, 4199–4208. 10.1021/acsnano.8b09198. 30883108

[ref45] Sugimoto H.; Fujii M. Colloidal Mie Resonant Silicon Nanoparticles. Nanotechnology 2021, 13, 4199–4208. 10.1088/1361-6528/ac1a44. 34343972

[ref46] Staude I.; Miroshnichenko A. E.; Decker M.; Fofang N. T.; Liu S.; Gonzales E.; Dominguez J.; Luk T. S.; Neshev D. N.; Brener I.; et al. Tailoring Directional Scattering through Magnetic and Electric Resonances in Subwavelength Silicon Nanodisks. ACS Nano 2013, 7, 7824–7832. 10.1021/nn402736f. 23952969

[ref47] Bogaerts W.; Selvaraja S. K.; Dumon P.; Brouckaert J.; De Vos K.; Van Thourhout D.; Baets R. Silicon-on-Insulator Spectral Filters Fabricated with CMOS Technology. IEEE J. Sel. Top. Quantum Electron. 2010, 16, 33–44. 10.1109/jstqe.2009.2039680.

[ref48] Gao N.; Chen M.; Xu H.; Xue Z.; Zhang N.; Fei L.; Wei X. Fabrication of Silicon-on-Insulator with High Uniform Top Si for Silicon Photonics Applications. Mater. Sci. Semicond. Process. 2020, 117, 105159 10.1016/j.mssp.2020.105159.

[ref49] Adachi S.; Taguchi T. Optical Properties of ZnSe. Phys. Rev. B 1991, 43, 9569–9577. 10.1103/physrevb.43.9569. 9996654

[ref50] Panda S. K.; Hickey S. G.; Demir H. V.; Eychmüller A. Bright White-Light Emitting Manganese and Copper Co-Doped ZnSe Quantum Dots. Angew. Chem. 2011, 50, 4432–4436. 10.1002/anie.201100464. 21480450

[ref51] Danek M.; Jensen K. F.; Murray C. B.; Bawendi M. G. Synthesis of Luminescent Thin-Film CdSe/ZnSe Quantum Dot Composites Using CdSe Quantum Dots Passivated with an Overlayer of ZnSe. Chem. Mater. 1996, 8, 173–180. 10.1021/cm9503137.

[ref52] Chen H.-S.; Lo B.; Hwang J.-Y.; Chang G.-Y.; Chen C.-M.; Tasi S.-J.; Wang S.-J. J. J. Colloidal ZnSe, ZnSe/ZnS, and ZnSe/ZnSeS Quantum Dots Synthesized from ZnO. J. Phys. Chem. B 2004, 108, 17119–17123. 10.1021/jp047035w.

[ref53] Wang X.; Zhu J.; Zhang Y. g.; Jiang J.; Wei S. One-Pot Synthesis and Optical Properties of Monodisperse ZnSe Colloidal Microspheres. Appl. Phys. A: Mater. Sci. Process. 2010, 99, 651–656. 10.1007/s00339-010-5692-2.

[ref54] Islam S. K.; Tamargo M.; Moug R.; Lombardi J. R. Surface-Enhanced Raman Scattering on a Chemically Etched ZnSe Surface. J. Phys. Chem. C 2013, 117, 23372–23377. 10.1021/jp407647f.

[ref55] Alessandri I.; Lombardi J. R. Enhanced Raman Scattering with Dielectrics. Chem. Rev. 2016, 116, 14921–14981. 10.1021/acs.chemrev.6b00365. 27739670

[ref56] Zhao L.; Sun C.; Tian G.; Pang Q. Multiple-Shell ZnSe Core-Shell Spheres and Their Improved Photocatalytic Activity. J. Colloid Interface Sci. 2017, 502, 1–7. 10.1016/j.jcis.2017.04.056. 28463683

[ref57] Blanco L. A.; García De Abajo F. J. Spontaneous Light Emission in Complex Nanostructures. Phys. Rev. B 2004, 69, 205414 10.1103/physrevb.69.205414. 15259724

[ref58] Guzatov D. V.; Klimov V. V. Optical Properties of a Plasmonic Nano-Antenna: An Analytical Approach. New J. Phys. 2011, 13, 053034 10.1088/1367-2630/13/5/053034.

[ref59] Mohammadi A.; Sandoghdar V.; Agio M. Gold Nanorods and Nanospheroids for Enhancing Spontaneous Emission. New J. Phys. 2008, 10, 105015 10.1088/1367-2630/10/10/105015.

[ref60] Gaponenko S. V.; Guzatov D. V.; Strekal N. D. Strong Selective Anti-Stokes Raman Scattering Enhancement in Plasmonics Using Photon Density of States Engineering. J. Phys. Chem. C 2021, 125, 27654–27660. 10.1021/acs.jpcc.1c08445.

