# Supplementary material for: Engineering of the Photon Local Density of States: Strong Inhibition of Spontaneous Emission near the Resonant and High-Refractive Index Dielectric Nano-objects
Source: J Phys Chem C Nanomater Interfaces. 2022 Mar 16;126(12):5691–700. doi: 10.1021/acs.jpcc.1c09844 (PMC9173691; doi:10.1021/acs.jpcc.1c09844)
Supplement: Supplementary file 1 — jp1c09844_si_001.pdf [file jp1c09844_si_001.pdf]

Supporting Information

Engineering of the Photon Local Density of States: Strong Inhibition of Spontaneous Emission near the Resonant and High-Refractive Index Dielectric Nano-objects

Alina Muravitskaya,[‡ †,] Artur Movsesyan,[§,||,#,*] Dmitry V. Guzatov,[¦] Anne-Laure Baudrion,[§] Pierre-Michel Adam,[§] Sergey V. Gaponenko,[‡] and Remi Vincent[§]*

[‡] *B.I. Stepanov Institute of Physics, National Academy of Sciences of Belarus, 68 Nezavisimosti Ave, Minsk 220072, Belarus*

[§] *Light, Nanomaterials & Nanotechnologies (L2n), CNRS EMR 7004, Université de Technologie de Troyes, 12 rue Marie Curie, 10004 Troyes Cedex, France*

[¦] *Yanka Kupala State University of Grodno, str. Ozheshko 22, Grodno 230023, Belarus*

Present Addresses:

[†]*Department of Physics & Mathematics, University of Hull, HU6 7RX Hull, United Kingdom*

[||] *Institute of Fundamental and Frontier Sciences, University of Electronic Science and Technology of China, Chengdu 610054, China*

[#] *Department of Physics and Astronomy, Ohio University, Athens OH 45701, USA*

E-mail: alina.muravitskaya@gmail.com; movsesyan@gmail.com

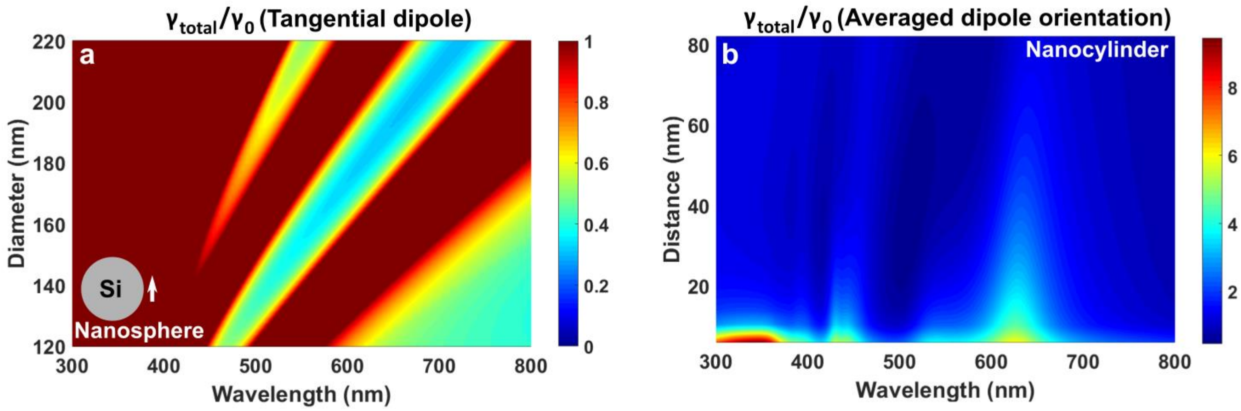

Figure S1. (a) Saturated map of the total decay rate of tangential dipole placed 10 nm far from the Si nanosphere in the air. (b) Calculated total decay rate map for the dipole (averaged orientations) placed at different distances from the silicon nanocylinder (NC) (180 nm diameter and 100 nm height) on the substrate.

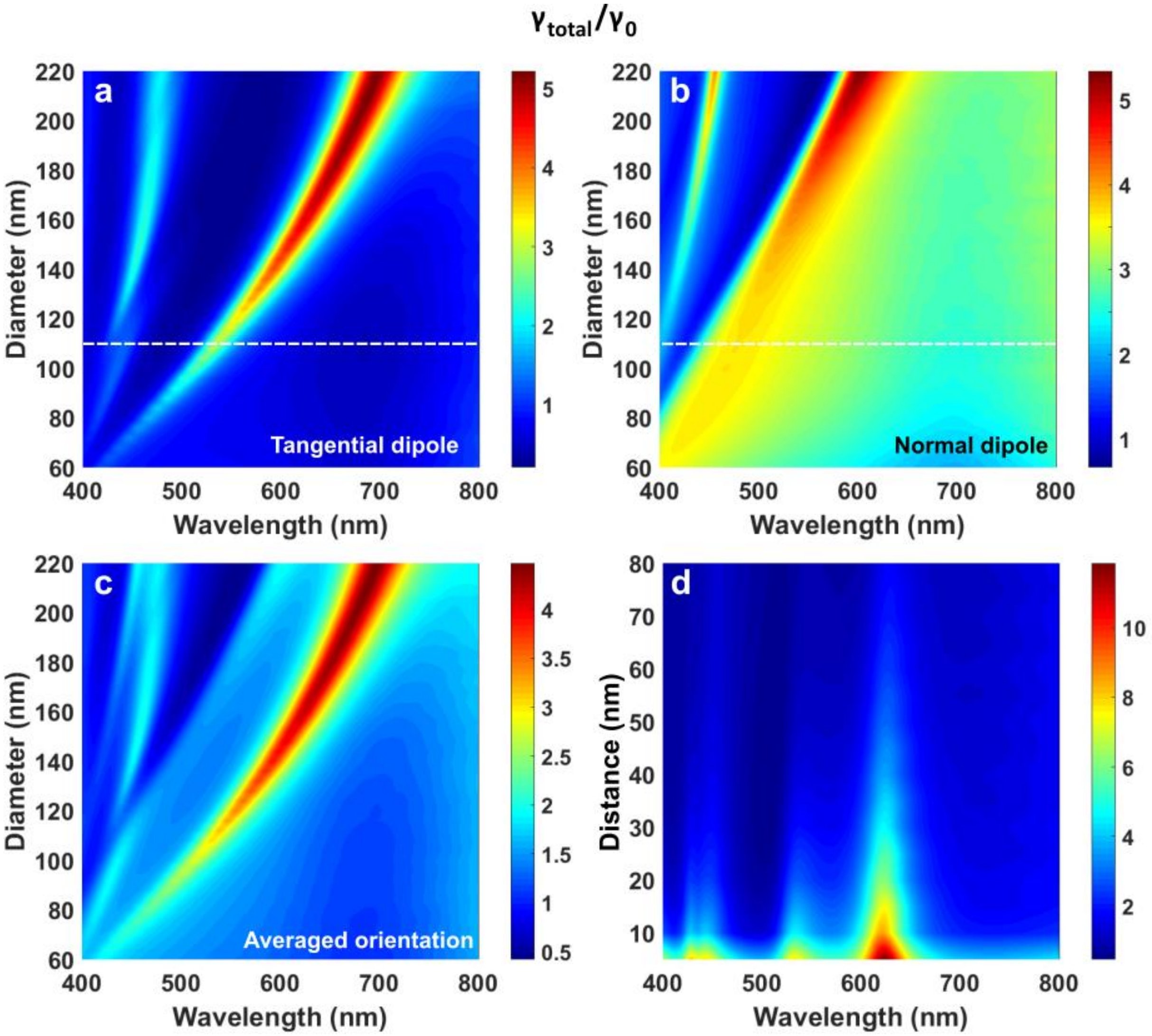

Figure S2. (a) Calculated total decay rate map for silicon nanocylinders of different diameters in the air excited by (a) tangential or (b) normal dipoles, and for averaged dipole orientation (c). Calculated total decay rate map for the dipole (averaged orientations) placed at different distances from the silicon NC in the air (180 nm diameter and 100 nm height) (d).

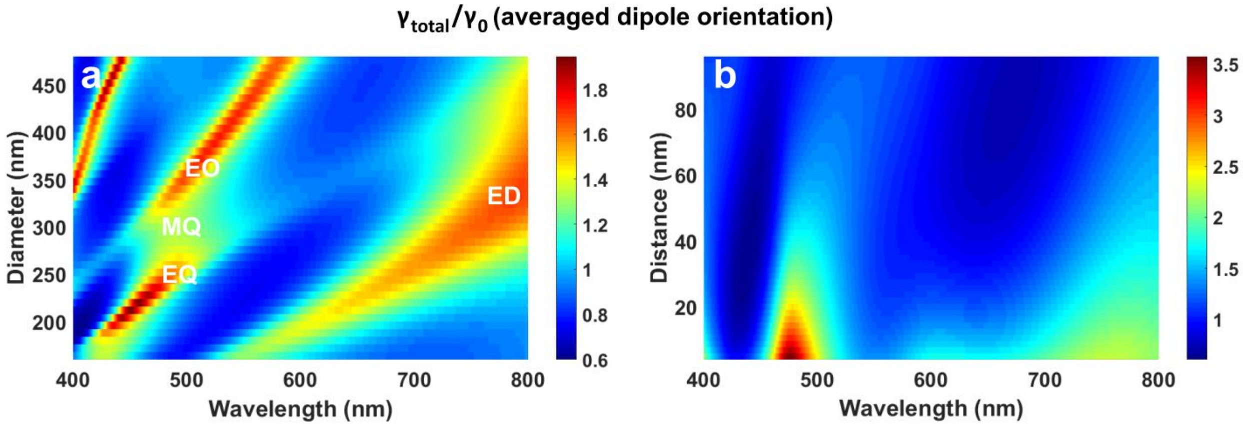

Figure S3. (a) Calculated total decay rate map for dipole (averaged orientations) near the ZnSe nanocylinder of 180 nm height and with different diameters on the substrate. The dipole is placed 35 nm far from the nanocylinder inside the substrate. (b) Calculated total decay rate map for the dipole (averaged orientations) placed at different distances from the 320 nm diameter and 180 nm height ZnSe nanocylinder.
